# Multilevel induction of apoptosis by microtubule-interfering inhibitors 4β-S-aromatic heterocyclic podophyllum derivatives causing multi-fold mitochondrial depolarization and PKA signaling pathways in HeLa cells

**DOI:** 10.18632/oncotarget.8147

**Published:** 2016-03-17

**Authors:** Ya-Xuan Zhang, Wei Zhao, Ya-Jie Tang

**Affiliations:** ^1^ Key Laboratory of Fermentation Engineering (Ministry of Education), Hubei Key Laboratory of Industrial Microbiology, Hubei Provincial Cooperative Innovation Center of Industrial Fermentation, Hubei University of Technology, Wuhan 430068, China

**Keywords:** podophyllum derivatives, carbon-sulfur and carbon-amine bonds, mitochondrial depolarization, antitumor mechanism

## Abstract

Herein is a first effort to study effect of carbon-sulfur (C-S) and carbon-nitrogen (C-N) bonds modification on the antitumor activity of the podophyllum derivatives in HeLa cells. Compared with the derivative modified by the C-N bond, the C-S bond modification exhibited superior antitumor activity by further causing significant mitochondria depolarization from three signaling pathway. First, a large number of microtubules were depolymerized by 4β-S-heterocyclic substituted podophyllum derivatives. The increasing free tubulin bond with voltage-dependent anion-selective channel (VDAC). Second, cAMP-dependent protein kinase A (PKA) was activated by 4β-S-heterocyclic substituted podophyllum derivatives. And then the activated PKA further caused significantly mitochondria depolarization. Third, the activated PKA also activated c-Jun N-terminal kinase (JNK) and further deceased MMP by improving the level of reactive oxygen species. Understanding the molecular events that contribute to drug-induced tumors apoptosis should provide a paradigm for a more rational approach to antitumor drug design.

## INTRODUCTION

Chemotherapy for the treatment of cancer was introduced into the clinic more than fifty years ago. Although this form of therapy has been successful for the treatment of some tumors, a causal relationship between tumor apoptosis and most of drug molecular structure has not been addressed. As the representative of the bioactive natural lead compound, podophyllotoxin (PTOX) and its analogue 4′-demethylepipodophyllotoxin (DMEP) is still a comparatively effective drug choice in the treatment of caner [1]. Numerous reviews emphasized the occurrence, synthesis and applications of PTOX, the recent progress towards development of structurally modified podophyllotoxin possessing apoptosis inducing ability [2]. With increasing the information about its structure-activity relationship (SAR) wide investigations have generated exciting chemotherapeutic candidates and successful applications of drug development from podophyllotoxin-related lead [3].

Tubulin was recently found to be a uniquely potent regulator of the voltage-dependent anion channel (VDAC) [4-6], the most abundant channel of the mitochondrial outer membrane, which constitutes a major pathway for ATP/ADP and other metabolites across this membrane [7]. Dimeric tubulin induces reversible blockage of VDAC reconstituted into a planar lipid membrane and dramatically reduces respiration of isolated mitochondria [5]. As tubulin promotes single-channel closure of VDAC, we hypothesized that tubulin is a dynamic regulator of ΔΨ, which in cultured cancer cells was assessed by flow cytometry (FCM) of the potential-indicating fluorophore tetramethylrhodamine methylester (TMRM) [8]. VDAC shows both ion selectivity and voltage dependence. In the open state, selectivity favoring anions over cations is weak [9]. VDAC shows both ion selectivity and voltage dependence. In the open state, selectivity favoring anions over cations is weak. Both positive and negative membrane potentials (50 mV) close VDAC [10]. It remains controversial if membrane potential activates VDAC conductance in intact cells. Nonetheless, VDAC closure effectively blocks movement of most organic anions, including respiratory substrates and creatine phosphate, and prevents exchange of ADP and Pi for ATP during oxidative phosphorylation[10]. Microtubule destabilizers colchicine, and nocodazole, and the microtubule stabilizer paclitaxel increased and decreased cellular free tubulin, respectively, and in parallel decreased and increased ΔΨ [11-14]. Protein kinase A (PKA) activation by cAMP analogues, whereas PKA inhibition hyperpolarized, consistent with reports that PKA decrease VDAC conductance, respectively [15-23].

In our previous work, compared with the derivative modified by carbon-nitrogen (C-N) bond, the derivative modified by carbon-sulfur (C-S) bond exhibited superior antitumor activity, the inhibition activity of target proteins tubulin or Topo II, cell cycle arrest, and apoptosis induction [24]. However, the antitumor mechanistic was unknown. In the present work, by taking podophyllum compound as an excellent research model for the natural lead compound, the research is to systematically study the precise apoptosis mechanism of the C-S and C-N bonds modification on the antitumor activity of podophyllum derivatives. The results can provide valuable information for other natural lead compounds and pave the way for rational drug design.

## RESULTS

### Apoptosis studies

**Figure 1:**
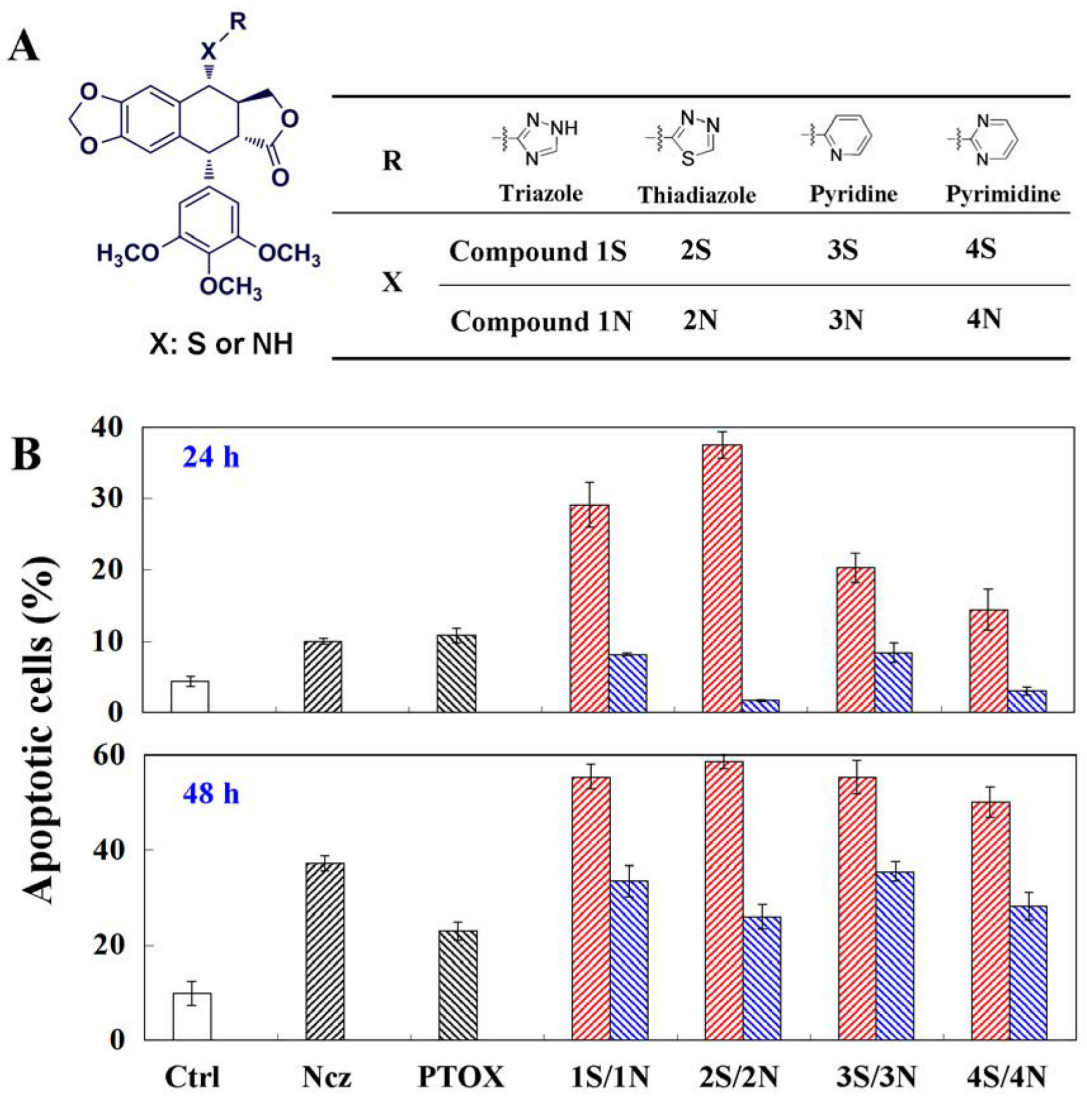
**A.** four couples respectively podophyllotoxin derivatives substituted by carbon-sulfur- and carbon-nitrogen-bond; **B.** Apoptosis detection in HeLa cells using annexin V and propidium iodide (PI) double staining after 24 and 48 h treatments of nocodazole, podophyllotoxin, and S series and N series compounds. Each value represents the mean ± SE of three independent experiments. *p <0.05. **p <0.01.

Apoptosis induced by the C-S bond modification podophyllum derivatives (S series compounds) and the C-N bond modification podophyllum derivatives (N series compounds) (Figure 1A) through depolymerizing microtubule in HeLa cells. S series compounds induce remarkable amounts of apoptosis of HeLa cells at 24 h, while N series not. However, when it comes to 48 h treatments of compounds, the differences of apoptosis between S and N series become inconspicuous. Compared to HepG_2_ and A549 cells, tumor cell HeLa exhibited the strong drug sensitivity to the 4β-*S* and 4β-*NH*-aromatic heterocyclic podophyllum derivative in the above *in vitro* cytotoxicity experiment. As a cell type in an immortal cell line, HeLa cells were often also used in the mechanism of antitumor drug scientific research. So, HeLa cells were used as a cell model for the following study. Notably, the cell cycle arrest ratio induced by Compound 1S was higher than Compound 1N throughout the 12-48 h. The primarily G_2_/M arrest noted at 24 and 48 h might be not consistent with the apoptosis. Following the treatment of Compounds 1S, 1N, 1′S and 1′N at the concentration of 0-5 μM for 6-48 h, the highest ratio up to 60% and 50% of cells were detected to be undergoing apoptosis, respectively. Interestingly, the C-S and C-N bonds modification aromatic heterocyclic podophyllum derivatives exhibited the similar effect on the G_2_/M phase arrest, but the apoptosis cells induced by Compound 1S were significantly higher than Compound 1N, and Compound 1′S showed higher potent than Compound 1′N to induce the cell death through apoptosis (Figure 1B). The above results demonstrated that the C-S bond modification aromatic heterocyclic podophyllum derivatives might induce apoptosis via an extraordinary mechanism.

### Mitochondrial membrane depolarisation and VDAC phosphorylation

**Figure 2:**
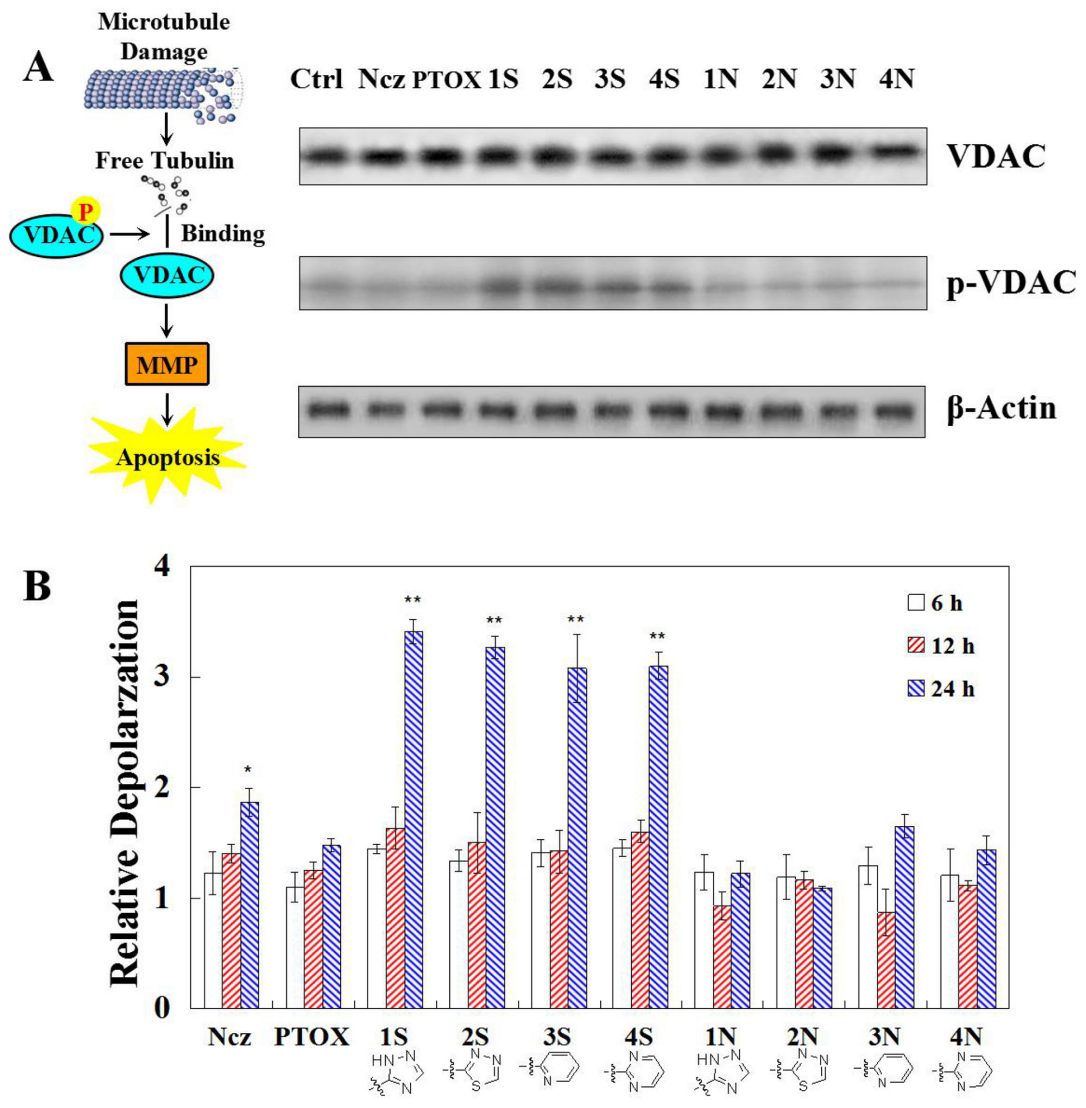
**A.** Total VDAC detected by Western blot and VDAC phosphorylation detected with phospho-stain after 12 h teartments of nocodazole, podophyllotoxin, and S series and N series compounds; **B.** Mitochondrial depolarization detection in HeLa cells using TMRM staining after 0-36 h treatment of nocodazole, podophyllotoxin, and S series and N series compounds. Each value represents the mean ± SE of three independent experiments. *p <0.05. **p <0.01.

Comparing with normal cells, Microtubule of treated cells depolymerized by colchine and polymerized by paclitaxel. S series compounds have higher microtubule depolymerizing ability against HeLa cells remarkably than N series. The expression of total VDAC remains substantially unchanged, after 12 h treatments of S and N series compounds. While only the S series compounds up-regulate the phosphated VDAC protein. S series compounds may induce MMP decreased by enhancing combinations of free tubulin and VDAC phosphorylation (Figure 2A). MMP decreased remarkably after treaments of S series compounds at 24 h. Compared with N series, S series compounds have higher ability of depolarzing HeLa cells remarkably (Figure 2B). N series compounds may not induce mitochondrial depolarizing for apoptosis.

### PKA activation detection Effects on VDAC phosphorylation of PKA inhibition and MMP of PKA inhibition

**Figure 3:**
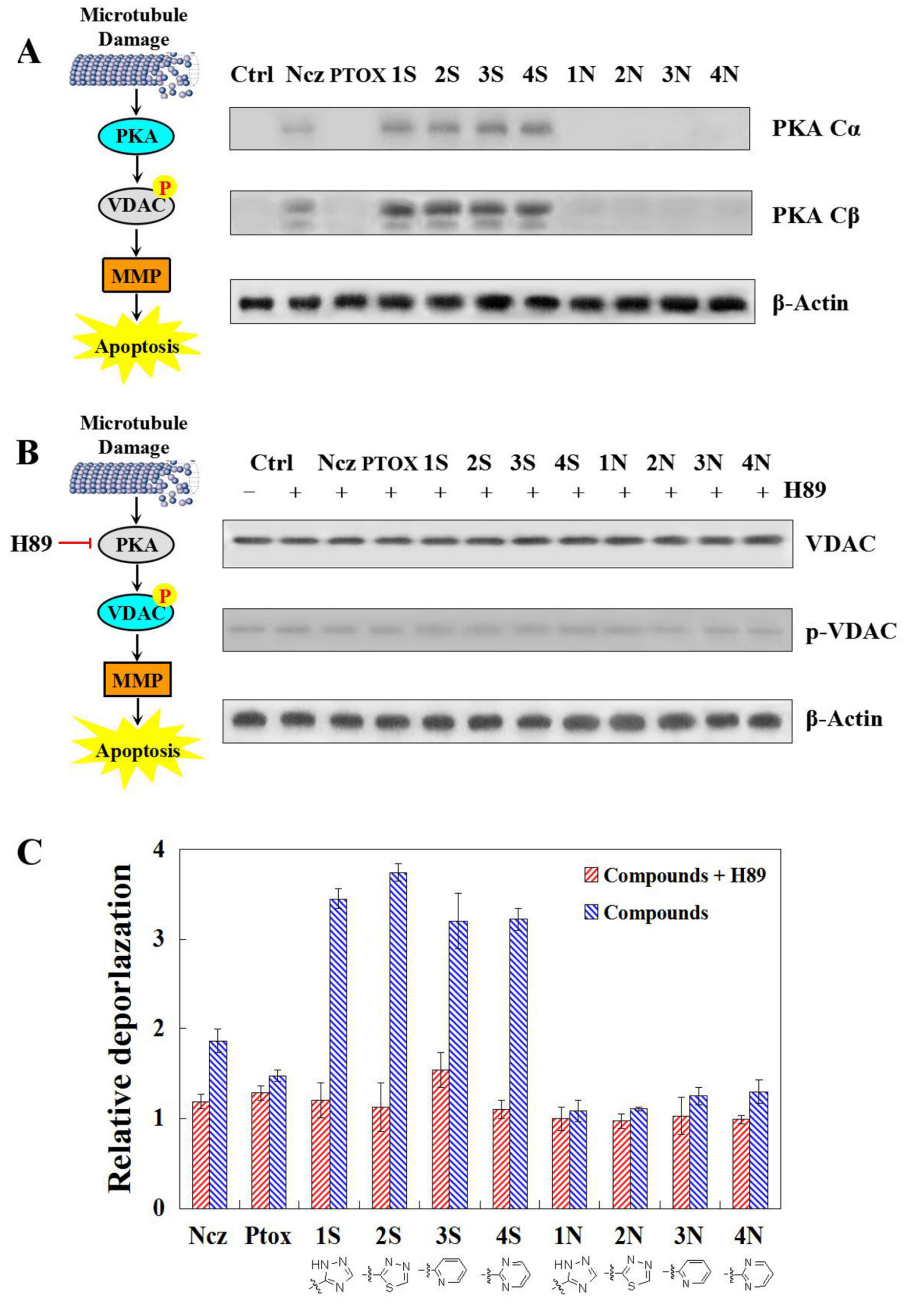
**A.** PKA catalytic subunits alpha and beta detected by Western blot after 6 h teartments of nocodazole, podophyllotoxin, and S series and N series compounds; **B.** VDAC phosphorylation detected with phospho-stain after 1 h per-teatments of PKA inhibitor H89 and then 12 h teartments of nocodazole, podophyllotoxin, and S series and N series compounds. **C.** Mitochondrial depolarization detection in HeLa cells using TMRM staining after 1 h per-teatments of PKA inhibitor H89 and then 24 h teartments of nocodazole, podophyllotoxin, and S series and N series compounds. Each value represents the mean ± SE of three independent experiments. *p <0.05. **p <0.01.

PKA cα subunit has been significantly activated by 12 compounds, especially S series, which effects better than N series in HeLa cells at 6 h. As previously reported, PTOX derivatives induce the apoptosis of cancer cells by damaging the spindle assemble in mitosis (Figure 3A). With the inhibitory effect against PKA activation of H89, S series compounds lose the ability of phosphorylating VDAC protein after 12 hours treatments. This shows that VDAC phosphorylation result from PKA activation induced by S series compounds (Figure 3B). Furthermore, after pre-treatment of H89 against HeLa cells, effects on MMP of S and N series compounds have been detected respectively after their 12 and 24 treatments. It turns out, the relative depolarization activated by these microtubule-damage agents at 12 hours remain unchanged basically before or after pre-treatment of PKA inhibitor. However, when the exposal time extends into 24 hours, the mitochondria depolarization induced by nocodazole and S series compounds have been inhibited obviously. Therefore, the slight MMP decrease caused by 6 hours treatments of microtubule-damage agents is PKA-independent which means that it may just results from the free tubulin and unphosphorylated VADC. Relatively, PKA activation induced by S series compounds more contributes to the effects on MMP of 12 hours treatments through VDAC phosphorylation (Figure 3C).

### ROS production detection and effects on MMP of ROS inhibition

**Figure 4:**
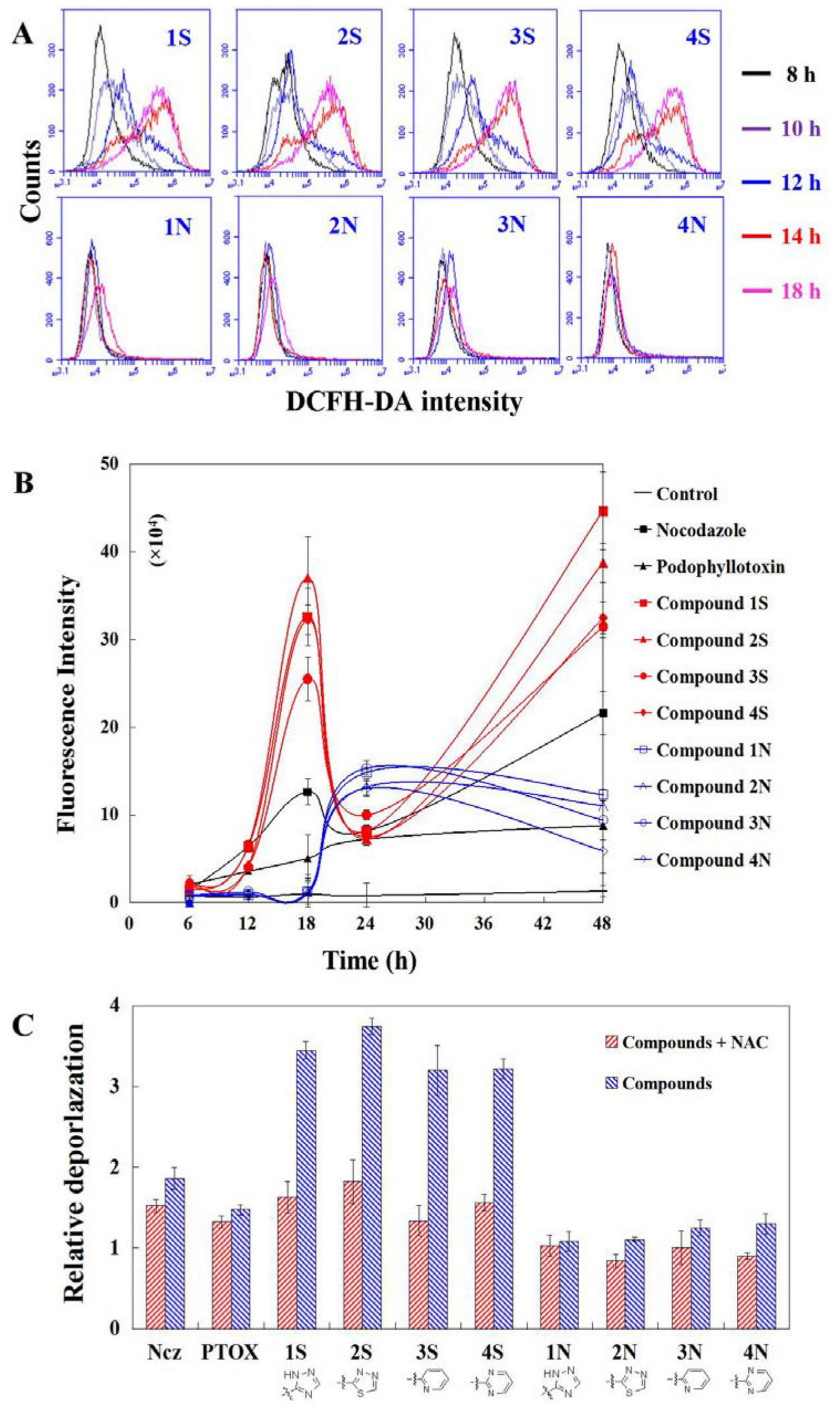
**A.** ROS production detection in HeLa cells using DCFH-DA staining after 8-18 h treatments of nocodazole, podophyllotoxin, and S series and N series compounds; **B.** Time-dependent manners of ROS production after 6-48 h treatments of nocodazole, podophyllotoxin, and S series and N series compounds; **C.** Mitochondrial depolarization detection in HeLa cells using TMRM staining after 1 h per-teatments of ROS inhibitor NAC and then 24 h teartments of nocodazole, podophyllotoxin, and S series and N series compounds. Each value represents the mean ± SE of three independent experiments. *p <0.05. **p <0.01.

ROS production caused by S series compounds is more and earlier than that of N series. ROS significantly induced by S series at 12 h in HeLa cells. Time-dependent detection shows that ROS production increase significantly after 14 h treatments of S series compounds, while that of N series start to rise at 18 h (Figure 4A). ROS inhibitor blocks depolarization induced by S series compounds. Mitochondria depolarization results from increased ROS caused by S series compounds in HeLa cells at 24 h. ROS production induced by S series compounds results in mitochondria depolarization for apoptosis (Figure 4B).

### Effects on ROS production and apoptosis of PKA inhibition

Mitochondria depolarization results from PKA activation caused by S series compounds in HeLa cells at 24 h. The increasing ROS production at 18 h results from PKA activation caused by S series compounds in HeLa cells. The increasing ROS production at 18 h results from PKA activation caused by S series compounds in HeLa cells. N series compounds may induce another apoptosic signal pathway different from S series (their lack of ability of depolymerizing MT and depolarzating cells). ROS are formed as a natural byproduct of the normal metabolism of oxygen and have important roles in cell signaling and homeostasis. However, excessive ROS can induce apoptosis through both the extrinsic and intrinsic pathways. In this study, a significant early increase of ROS in cells were induced by Compound 1S and Compound 1N compared to the control (the cells incubated with no drug) after 6 and 12 h. After 24 h of induction with Compound 1S and Compound 1N, a rapid increase of the fluorescence intensity of ROS was observed. The fluorescence intensity of ROS further increased from 10^5^−10^6^ to 10^6^−10^7.2^ for a half of cells after 48 h induction with Compound 1S. While, the fluorescence intensity of ROS decreased from 10^5^-10^6^ to 10^4^−10^5^ for the most part cells after 48 h induction with Compound 1N (Figure 4C). Compared with the Compound 1N, the amount of ROS (calculated as: the fluorescence intensity of ROS × the count of cells containing ROS) induction with Compound 1S were improved by 4.8 times after 48 h. The above results demonstrated that the C-S bond modification aromatic heterocyclic podophyllum derivatives showed the higher potency for generating ROS than those of the C-N bond in HeLa cells (Figure 4C). And this is consistent with Compound 1N being less efficient and weaker than Compound 1S on the activated caspase-9 in the mitochondrial apoptotic pathways.

## DISCUSSION

Sulfur and nitrogen atom exist in natural products and clinical drugs widely, they made a significant contribution for the balance of heteroatom substituent in drugs molecules. This bioisosteric replacement may dramatically change the biological activity of the natural lead compound. Based on four couples respectively podophyllotoxin derivatives substituted by C-S and C-N bond as object, we explore the specific molecular mechanism of difference between their effects on anticancer activity against human cervical carcinoma HeLa cells. It turns out S-series compounds substituted by C-S bond have higher depolymerization ability on their target microtubule, which is superior to N-series compounds substituted by C-N bond.

**Figure 5:**
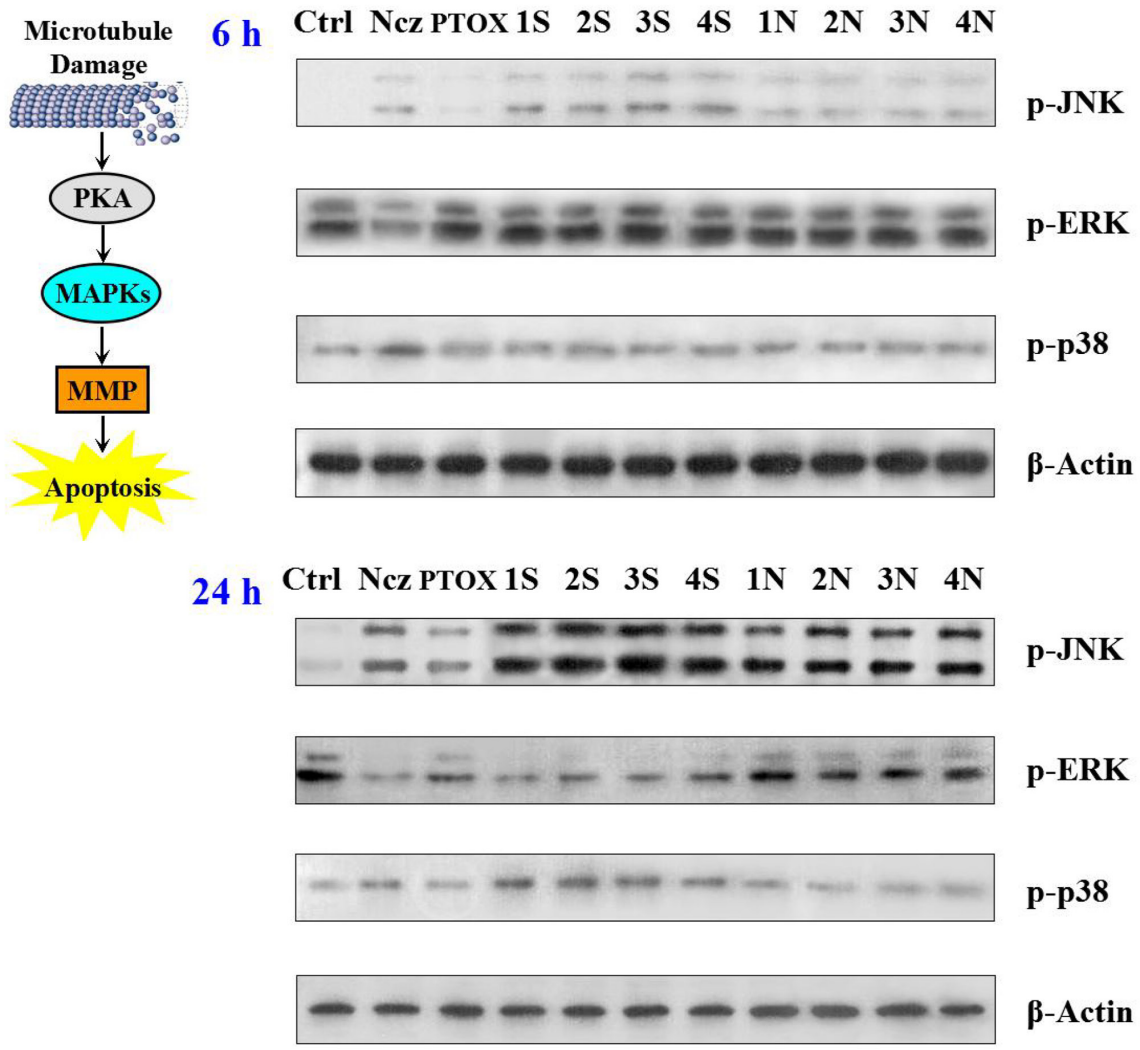
Effect of nocodazole, podophyllotoxin, and S series and N series compounds on the levels of the MAPKs, JNK; ERK; p38 and their phosphorylated forms using Western blot analysis after 6 and 24 h treatments

**Figure 6:**
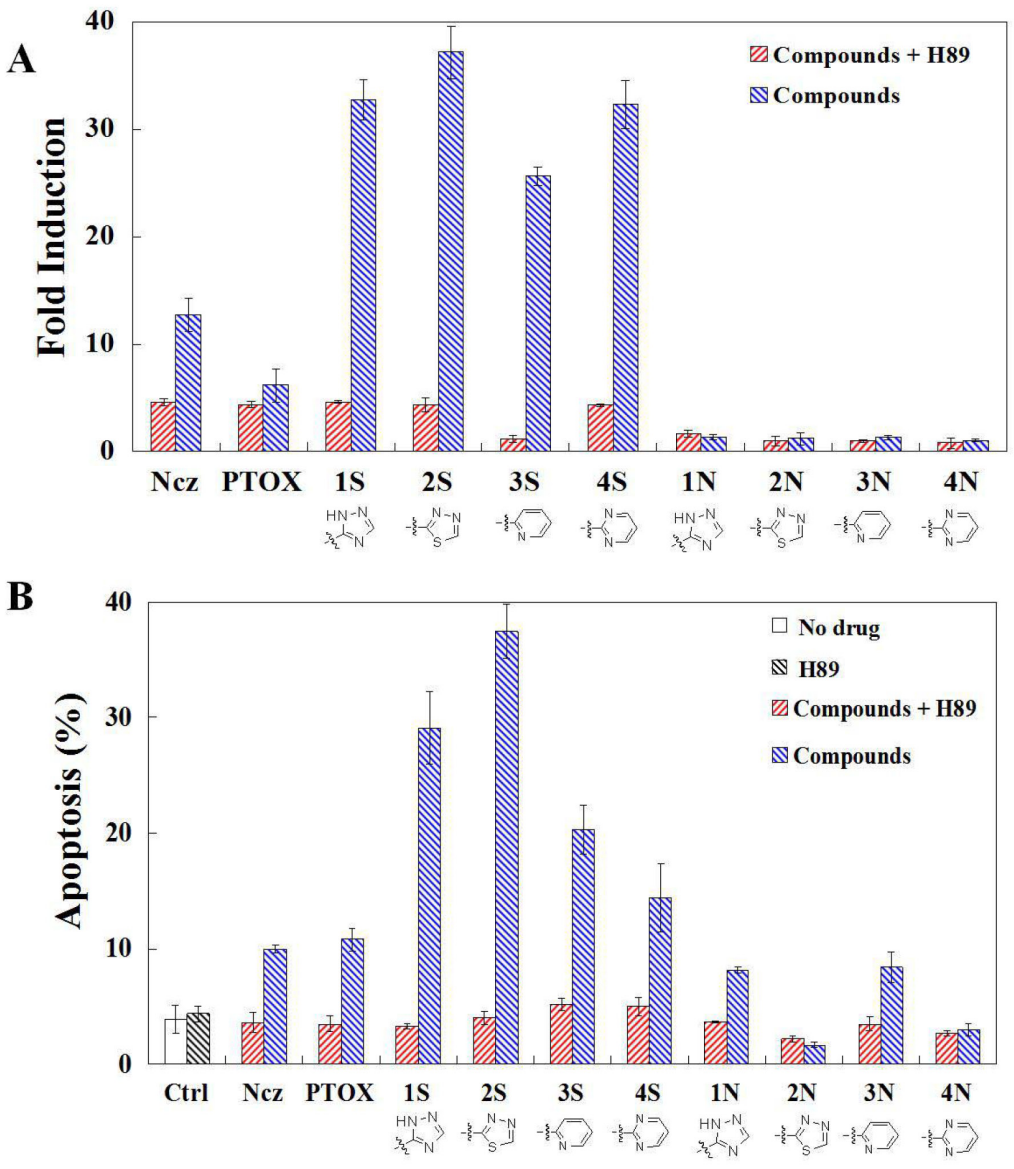
**A.** ROS production detection in HeLa cells using DCFH-DA staining after 1 h per-teatments of PKA inhibitor H89 and then 24 h teartments of nocodazole, podophyllotoxin, and S series and N series compounds; **B.** Apoptosis detection in HeLa cells using annexin V and propidium iodide (PI) double staining after 1 h per-teatments of PKA inhibitor H89 and then 24 h teartments of nocodazole, podophyllotoxin, and S series and N series compounds. Each value represents the mean ± SE of three independent experiments. *p <0.05. **p <0.01.

**Figure 7:**
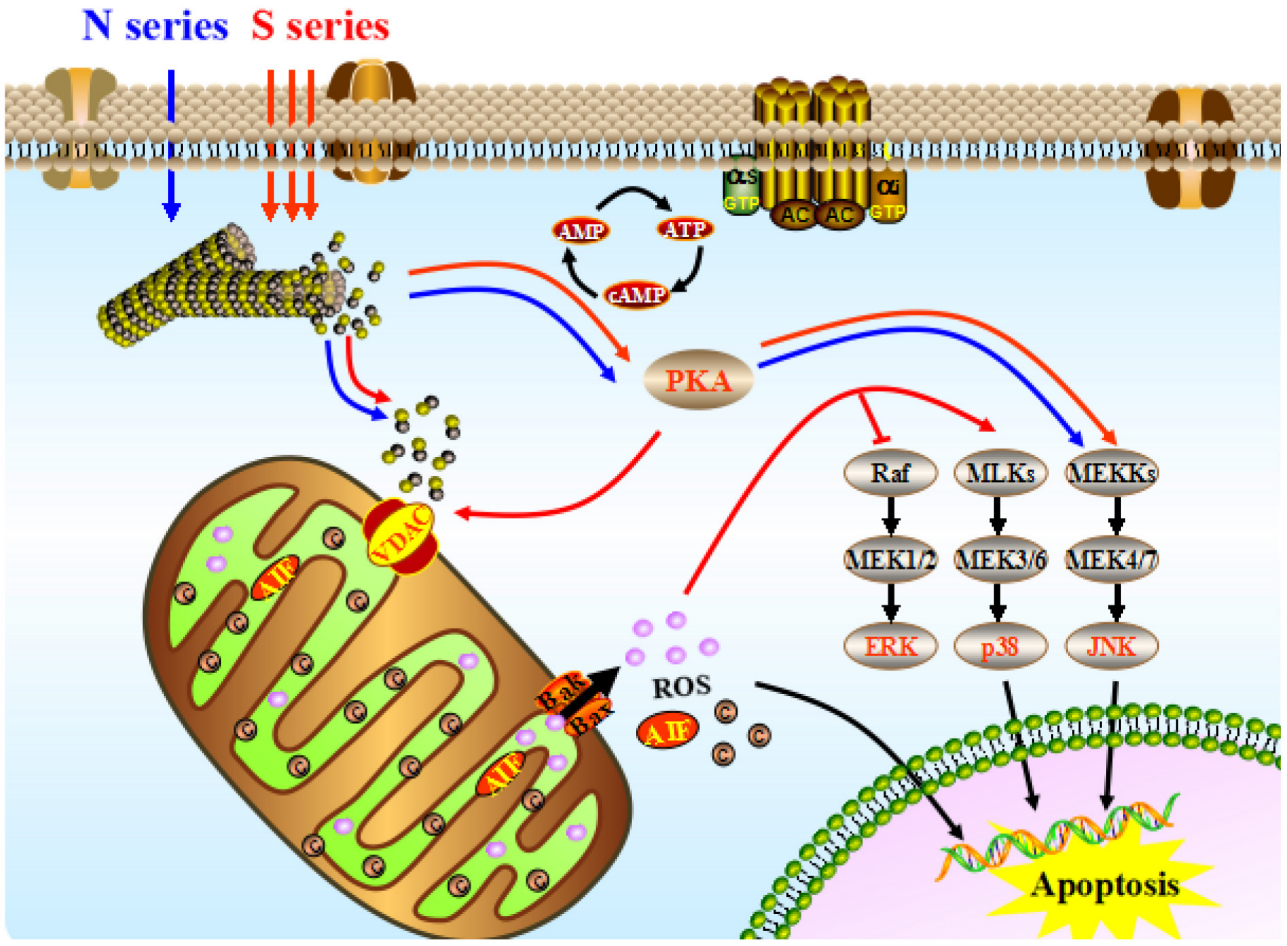
The integrated apoptotic pathways, a schematic diagram showing some of the known components of the intrinsic and the death receptor apoptotic programs and the mitochondrial apoptotic pathways Symbols: the pathways were activated by S series compounds (→); the pathways were activated N series compounds (→).

The first round of signal regulation in HeLa cells is induced by the activation of PKA. Meanwhile, the direct effector of second messenger cAMP, PKA is activated by S-series compounds after 6 hours treatment. On the one hand, VDAC is phosphorylated to enhance its combination with free tubulin leading to mitochondria depolarization. On the other hand, JNK signaling pathway is activated to promote apoptosis in MAPK (mitogen-activated protein kinases) cascade reaction (Figure 5). Phosphorylated JNK results in MMP critical decreasing and further mitochondrial dysfunction by inducing a mass of increasing ROS production. Massive ROS from mitochondria released into cytoplasm begin to switch second round of signal regulation through activating p38 MAPK-mediated apoptosis signaling and inhibiting ERK-mediated in growth and survival signaling at the same time, leading to HeLa cells apoptotic death. However, comparing with S series compounds, N series compounds just activate the first round of signal regulation after 12-hours treatments, which is much later (Figure 6). As a result of their lower cellular uptake, N series compounds are impeded getting into HeLa cells to play their antitumour roles [24, 25]. The electronegativity of sulfur atom is lower than that of nitrogen atom, the hydrophobic thioether bond (−S-) is better than imino bond (-NH-) to improve the cellular uptake of the compounds which may enhance the biological activity. N series compouds with slight microtubule depolymerizing ability induce mitochondrial apoptosis in HeLa cells by acvtivating PKA signaling after treatments delay (Figure 7). Most of the currently available antitumor drugs have been discovered empirically by screening of large numbers of compounds for efficacy against tumor models[26-28]. The rational design of a drug is usually based on biochemical and physiological differences in tumor. Herein, the difference between the carbon-sulfur (C-S) and carbon-amine (C-N) bond modification on the antitumor activity of natural lead compound podophyllum derivatives and their precise mechanism were systematically studied for the first time.

## MATERIALS AND METHODS

### Inmunofluorescence

HeLa cells were continuously maintained in DMEM medium supplemented with 12% fetal calf serum (FCS), 2 mM/L -glutamine, and 100 U/mL penicillin and streptomycin at 37°C and 5% CO_2_. HeLa cells (200000 per mL) were plated a onto 6-well tissue culture plates containing 12 mm round coverslips, cultured overnight, and then treated with drugs at different concentrations or drug vehicle (0.1% DMSO) for 24 h. Attached cells were permeabilized. Cytoskeletons were incubated with α-tubulin, washed twice, and incubated with FITC goat anti-mouse immunoglobulins. The coverslips were washed, and 1 μg/mL DAPI to stain chromatin was added. The mixture was incubated for 30 min. After the samples were washed, they were examined and photographed using an Olympus epifluorescence microscope. The images were recorded with a Hamamatsu 4742-95 cooled CCD camera.

### Cell apoptosis analysis

The HeLa cell line was used for cell apoptosis. Cells (20000 per mL) were incubated with several concentrations of the compounds or drugs for 6-48 h, and incubated in DMEM medium supplemented with 12% fetal calf serum (FCS), 2 mM L -glutamine, and 100 U/mL penicillin and streptomycin at 37°C and 5% CO_2_. The cells were washed with PBS twice, centrifuged at 1500 rpm for 5 min, and 5-10^5^ cells were collected. Binding buffer suspension (500 μL) was added to the cells, and then 5 μL of the FITC-Annexin V mix was added. Next, 5 μL of the PI mix was added, and the suspension was mixed and kept at room temperature for 30 min in the dark. Analysis was with a BD accur C6 flow cytometer.

### Western blot analysis

For electrophoresis, the proteins were separated by sodium dodecyl sulfate–polyacrylamide gel electro-phoresis (SDS–PAGE). The proteins were then transferred to a nitrocellulose membrane, which was blocked with 5% skimmed milk in phosphate buffered saline Tween-20 (PBST). A speciffc primary antibody was added to bind the target proteins for either 1 h at room temperature or overnight at 4°C. A horseradish peroxidase (HRP) conjugated secondary antibody was added to the membrane after the primary antibody was washed off. All signals were detected after the HRP was activated by enhanced chemiluminescence.

### ROS production analysis

Changes in intracellular ROS levels were determined by measuring the oxidative conversion of cell permeable 2′,7′-dichlorofluorescein diacetate (DCFH-DA) to fluorescent di-chlorofluorescein (DCF) in flow cytometry (BD Accuri™ C6). Cells in 6-well culture dishes were incubated with DMEM for 6, 12, 24 and 48 h in the absence or presence of test compounds. The cells were washed with DMEM and incubated with DCFH-DA (10 μM) at 37°C for 30 min. Then DCF fluorescence of 10000 cells was detected by flow cytometry.

### Mitochondrial membrane depolarisation assay

Add 100 nM Tetramethylrhodamine, methyl ester (TMRM) directly to cells (5×10^5^/ml) in DMEM. Incubate the cells for 5 min at 37°C and measure the level of fluorescence in cells by flow cytometry using appropriate excitation and emission filters. TMRE is optimally excited at 549 nm and emits at 574 nm but can also be excited using a 488 nm laser available on most flow cytometers.

### Phosphorylation detection

Phosphorylation of VDAC was detected using Pro-Q Diamond phospho-protein stain (Invitrogen). The bands were imaged at 532–560 nm excitation on a Fuji gel scanner, and the difference in staining between control and phosphorylated VDAC was quantified using “Multi Gauge V3.0” software. The VDAC band intensities with gel background subtracted were normalized versus intensity of the untreated samples in each corresponding gel. All gels were analyzed in raw, unmodified state.

### Statistical analysis

Results are expressed as mean standard error (±SE) of means (SE) for separate groups. Group means SE were compared by one-way analysis of variance (ANOVA) followed by Turkey's post hoc test. Probability, p < 0.05, 0.01 was considered statistically significant.
